# Supplementary material for: Insights from the Endophytic Fungi in Amphisphaeria (Sordariomycetes): A. orixae sp. nov. from Orixa japonica and Its Secondary Metabolites
Source: Microorganisms. 2023 May 11;11(5):1268. doi: 10.3390/microorganisms11051268 (PMC10221786; doi:10.3390/microorganisms11051268)
Supplement: Supplementary file 1 [file microorganisms-11-01268-s001.zip › microorganisms-2357812-supplementary.pdf]

Supporting Information

Insights from the Endophytic Fungi in *Amphisphaeria* (Sordariomycetes): *A. orixae* sp. nov. from *Orixa japonica* and Its Secondary Metabolites

Xiaojie Wang [1,2], Dhanushka N. Wanasinghe [3], Jingyi Zhang [4], Jian Ma [4], Peifeng Zhou [1,2], Lijuan Zhang [4], Yongzhong Lu [4,*] and Zhen Zhang [2,*]

[1] School of Liquor and Food Engineering, Guizhou University, Guiyang 550025, China
[2] Guizhou Academy of Testing and Analysis, Guizhou Academy of Sciences, Guiyang 550014, China
[3] Centre for Mountain Futures, Kunming Institute of Botany, Chinese Academy of Sciences, Honghe County 654400, China
[4] School of Food and Pharmaceutical Engineering, Guizhou Institute of Technology, Guiyang 550003, China
* Correspondence: yzlu@git.edu.cn (Y.L.); zhangzhen@gzata.cn (Z.Z.)

List of Supporting Information

Figure S1. ^{1}H NMR spectrum of compound **1** in CD_3OD

Figure S2. ^{13}C NMR spectrum of compound **1** in CD_3OD

Figure S3. HRESIMS spectrum of compound **1**

Figure S4. IR spectrum of compound **1**

Figure S5. UV spectrum of compound **1**

Figure S6. HSQC spectrum of compound **1** in CD_3OD

Figure S7. HMBC spectrum of compound **1** in CD_3OD

Figure S8. ^{1}H-^{1}H COSY spectrum of compound **1** in CD_3OD

Figure S9. ^{1}H NMR spectrum of compound **2** in CD_3OD

Figure S10. ^{13}C NMR spectrum of compound **2** in CD_3OD

Figure S11. ^{1}H NMR spectrum of compound **3** in DMSO-d_6

Figure S12. ^{13}C NMR spectrum of compound **3** in DMSO-d_6

Figure S13. ^{1}H NMR spectrum of compound **4** in DMSO-d_6

Figure S14. ^{13}C NMR spectrum of compound **4** in DMSO-d_6

Figure S15. ^{1}H NMR spectrum of compound **5** in $CDCl_3$

Figure S16. ^{13}C NMR spectrum of compound **5** in $CDCl_3$

Figure S17. ^{1}H NMR spectrum of compound **6** in $CDCl_3$

Figure S18. ^{13}C NMR spectrum of compound **6** in $CDCl_3$

Figure S19. ^{1}H NMR spectrum of compound **7** in $CDCl_3$

Figure S20. ^{13}C NMR spectrum of compound **7** in $CDCl_3$

Figure S21. ^{1}H NMR spectrum of compound **8** in CD_3OD

Figure S22. ^{13}C NMR spectrum of compound **8** in CD_3OD

Figure S23. ^{1}H NMR spectrum of compound **9** in $CDCl_3$

Figure S24. ^{13}C NMR spectrum of compound **9** in $CDCl_3$

Figure S25. ^{1}H NMR spectrum of compound **10** in CD_3OD

Figure S26. ^{13}C NMR spectrum of compound **10** in CD_3OD

Figure S27. ^{1}H NMR spectrum of compound **11** in CD_3OD

Figure S28. ^{13}C NMR spectrum of compound **11** in CD_3OD

Figure S29. ^{1}H NMR spectrum of compound **12** in CD_3OD

Figure S30. ^{13}C NMR spectrum of compound **12** in CD_3OD

Figure S31. ^{1}H NMR spectrum of compound **13** in $CDCl_3$

Figure S32. ^{13}C NMR spectrum of compound **13** in $CDCl_3$

Figure S33. ECD calculation details of compound **1**

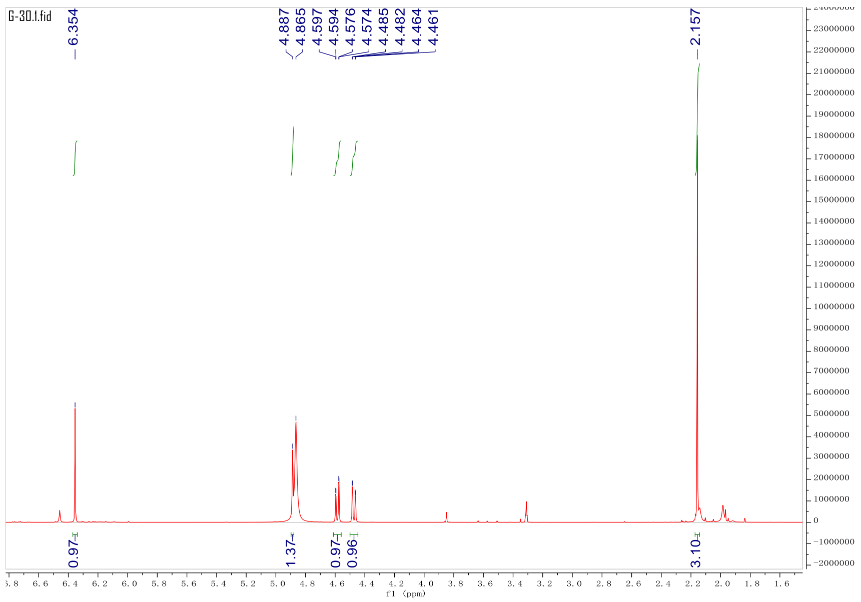

Figure S1. ^{1}H NMR spectrum of compound **1** in CD_3OD

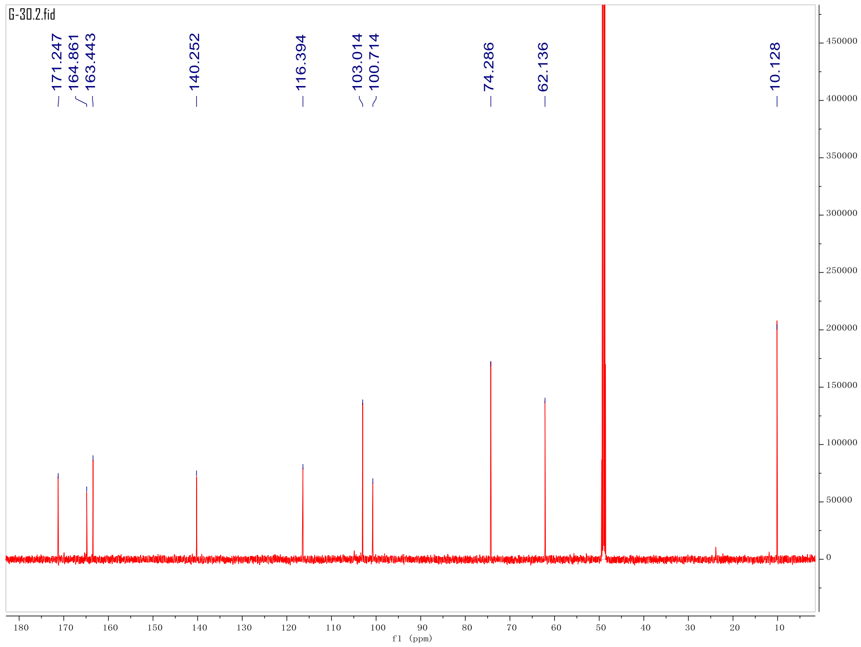

Figure S2. ^{13}C NMR spectrum of compound **1** in CD_3OD

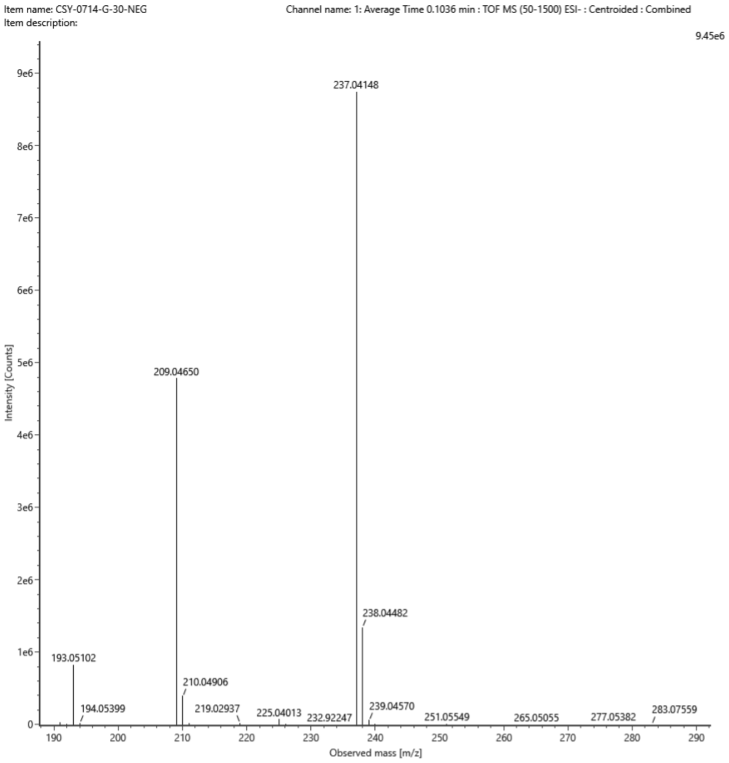

Figure S3. HRESIMS spectrum of compound **1**

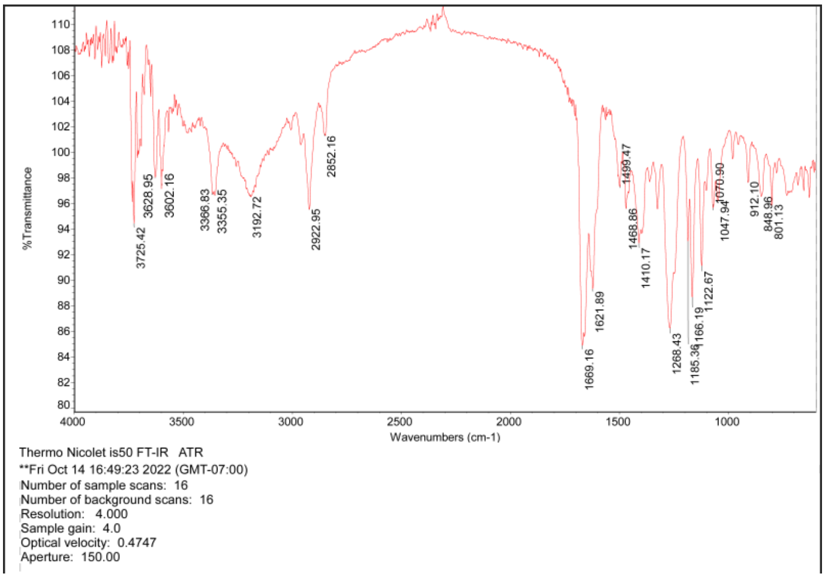

Figure S4. IR spectrum of compound **1**

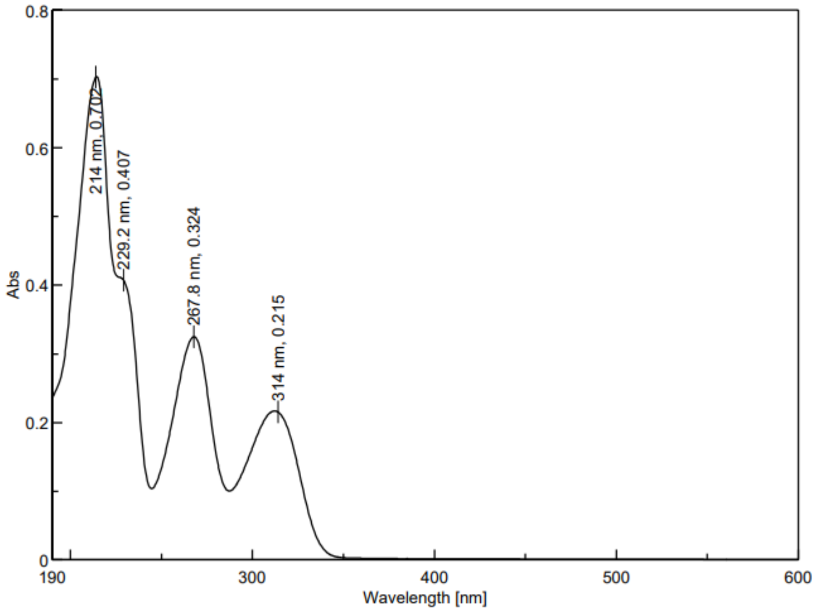

Figure S5. UV spectrum of compound **1**

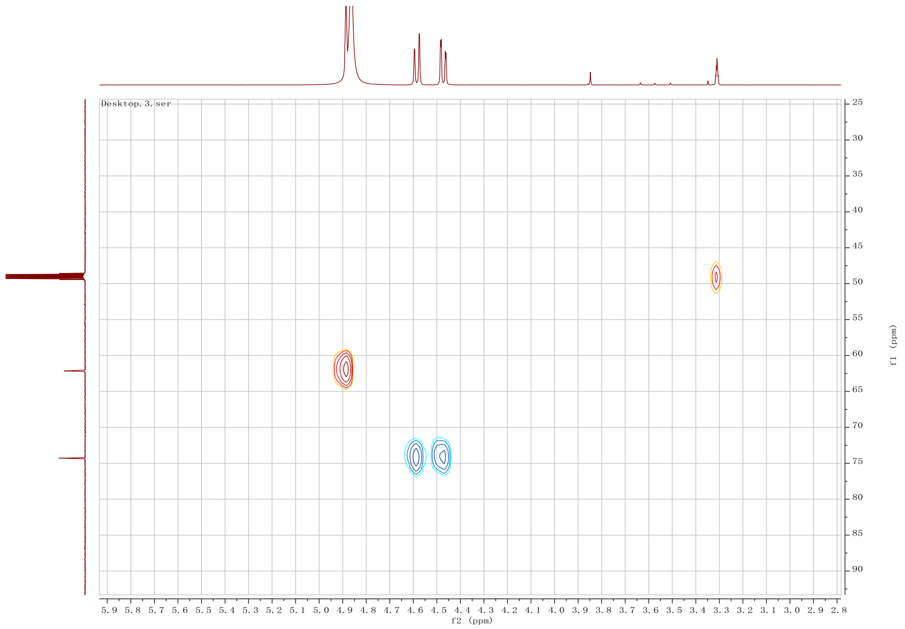

Figure S6. HSQC spectrum of compound **1**

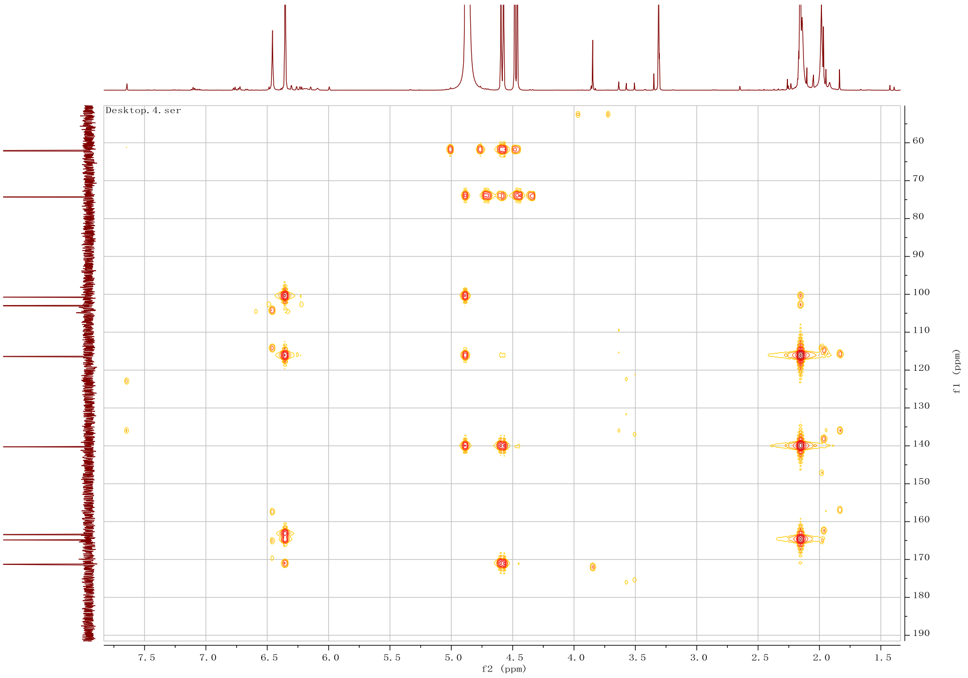

Figure S7. HMBC spectrum of compound **1**

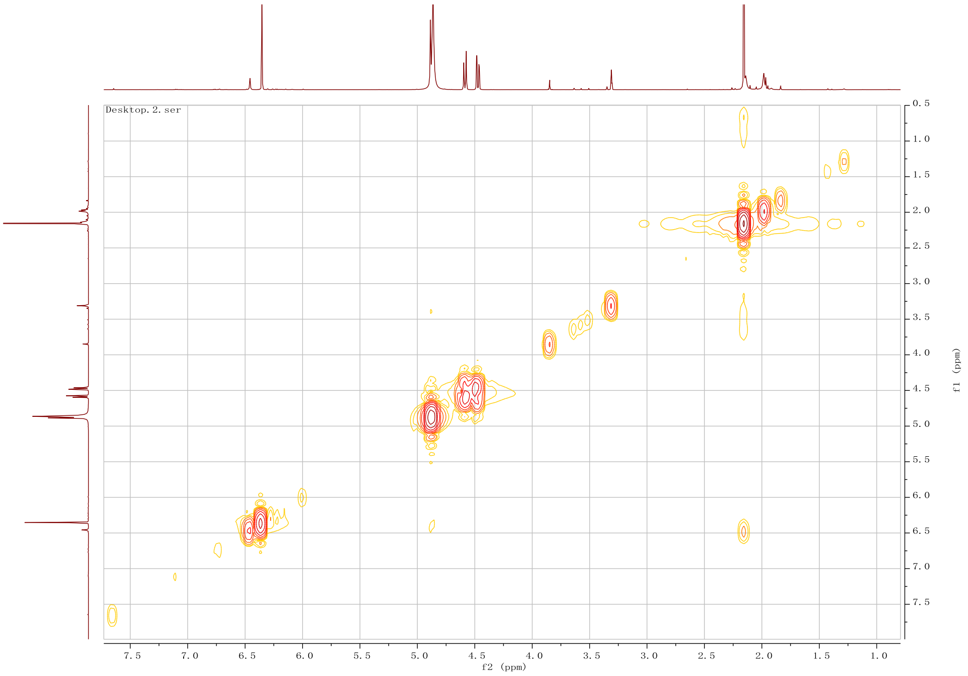

Figure S8. ^{1}H-^{1}H COSY spectrum of compound **1**

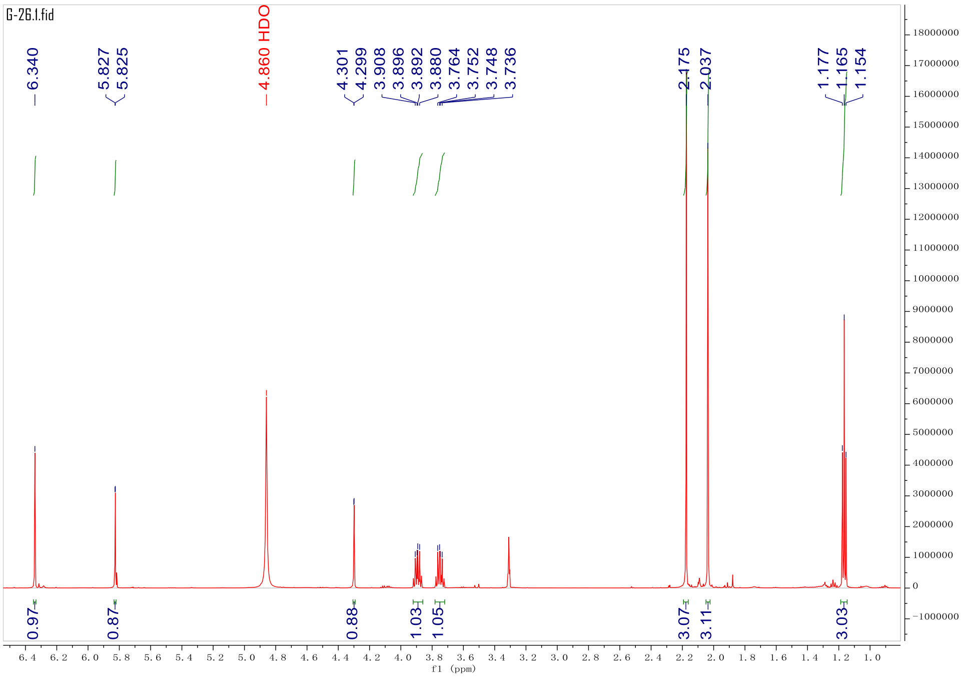

Figure S9. ^{1}H NMR spectrum of compound **2** in CD_3OD

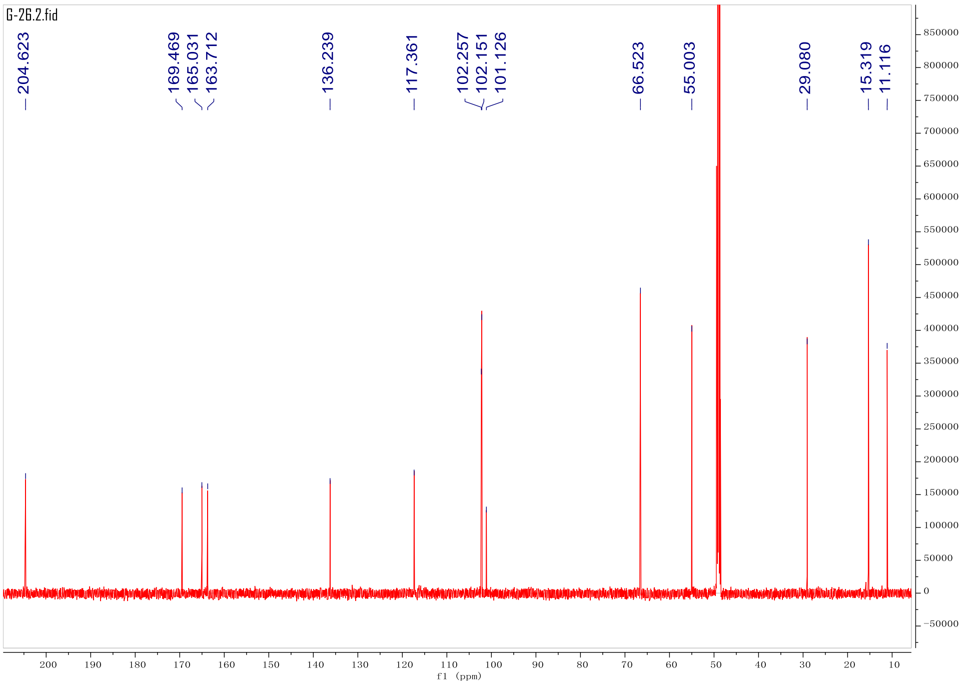

Figure S10. ^{13}C NMR spectrum of compound **2** in CD_3OD

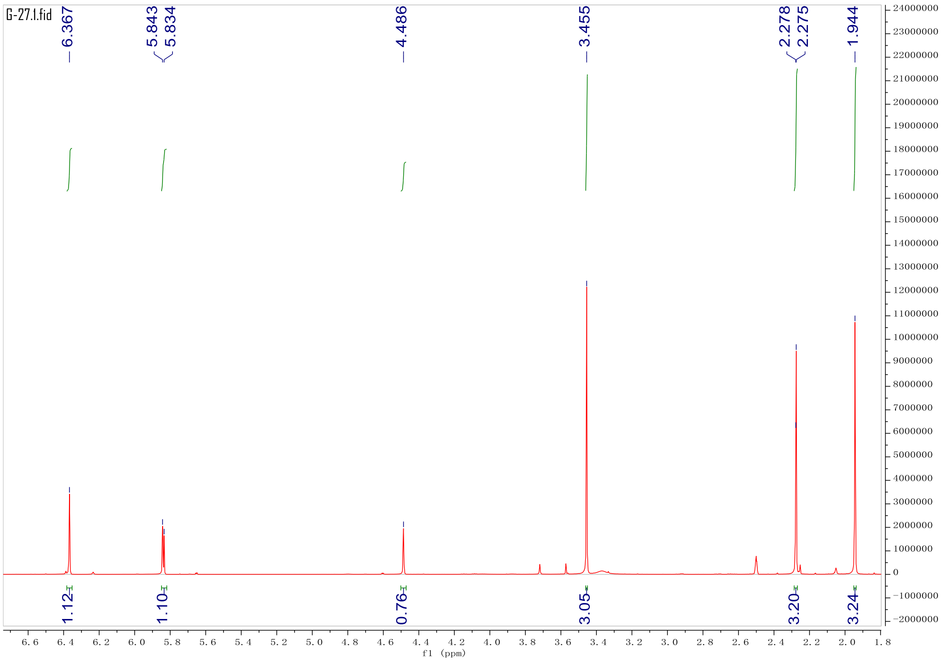

Figure S11. ^{1}H NMR spectrum of compound **3** in DMSO-d_6

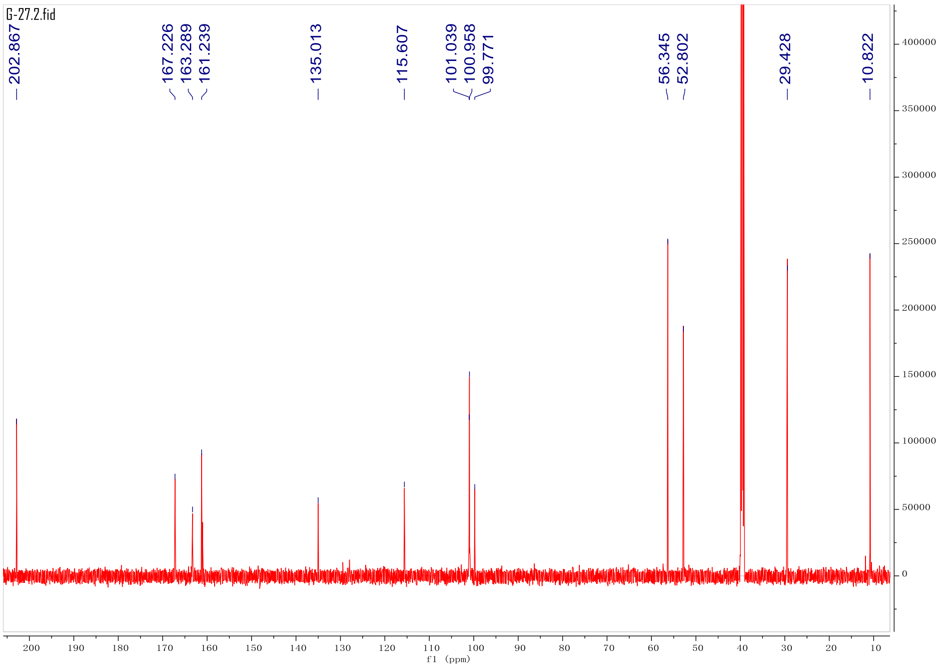

Figure S12. ^{13}C NMR spectrum of compound **3** in DMSO-d_6

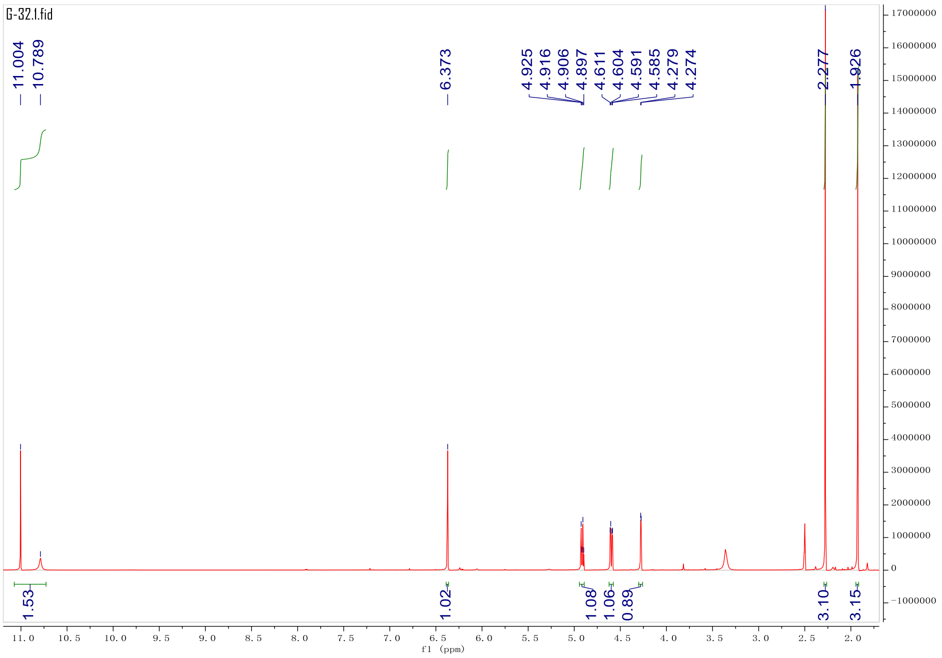

Figure S13. ^{1}H NMR spectrum of compound **4** in DMSO-d_6

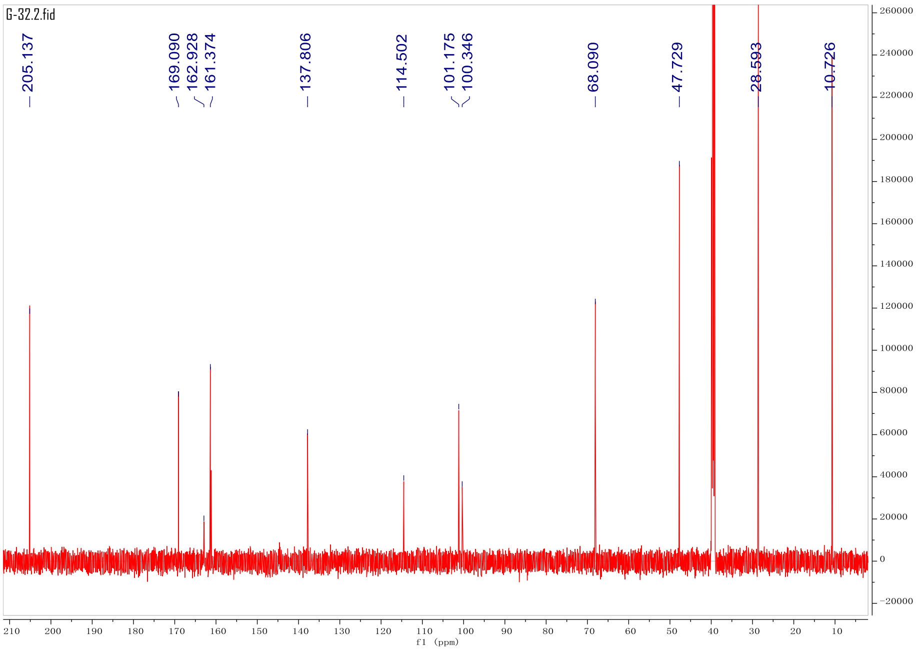

Figure S14. ^{13}C NMR spectrum of compound **4** in DMSO-d_6

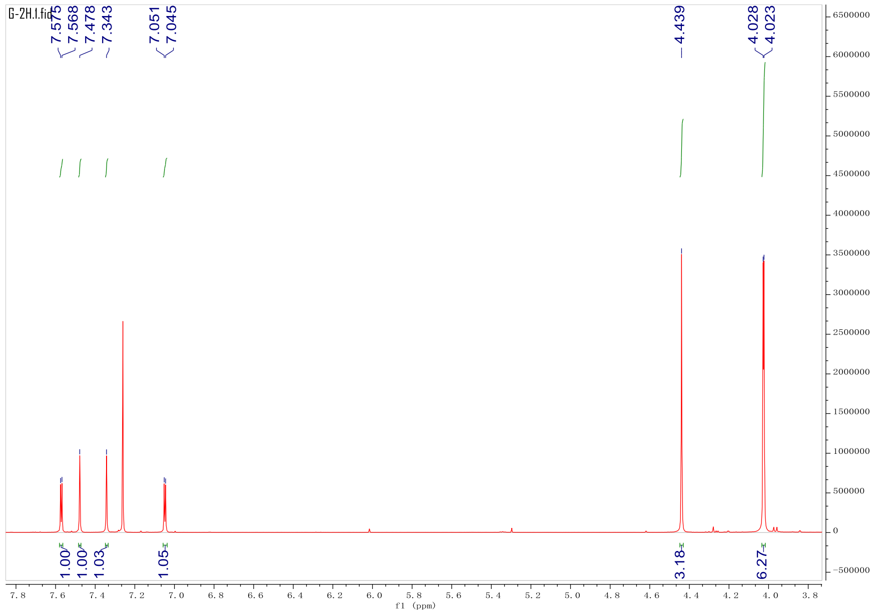

Figure S15. ^{1}H NMR spectrum of compound **5** in $CDCl_3$

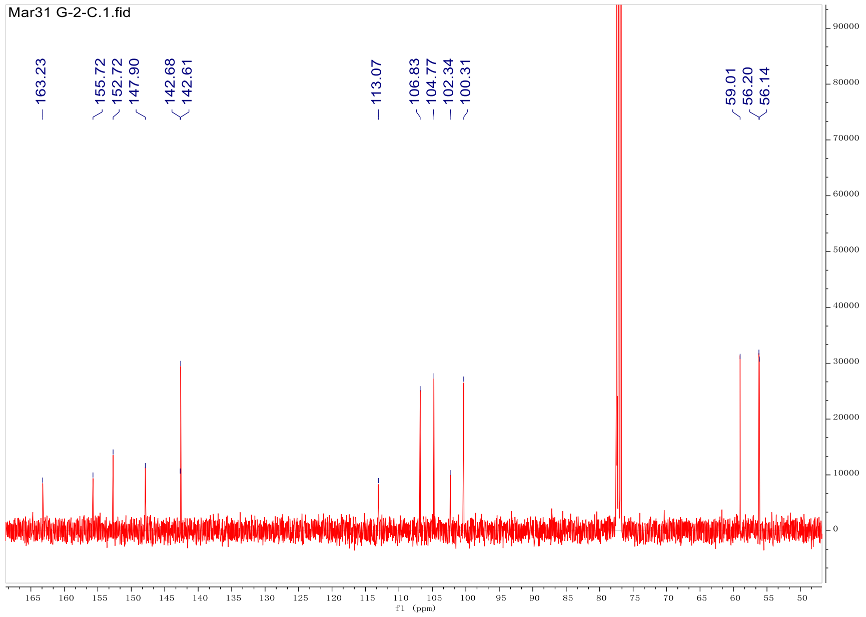

Figure S16. ^{13}C NMR spectrum of compound **5** in $CDCl_3$

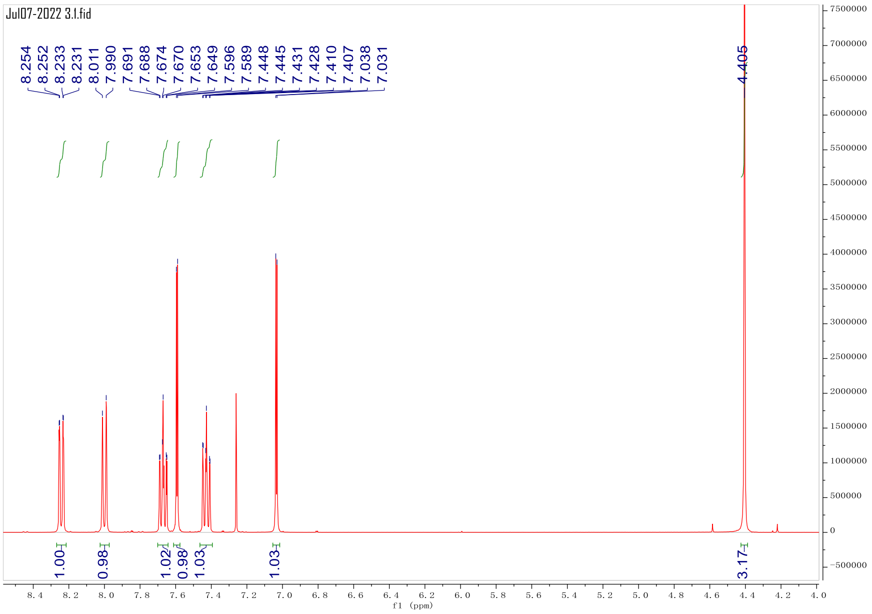

Figure S17. ^{1}H NMR spectrum of compound **6** in $CDCl_3$

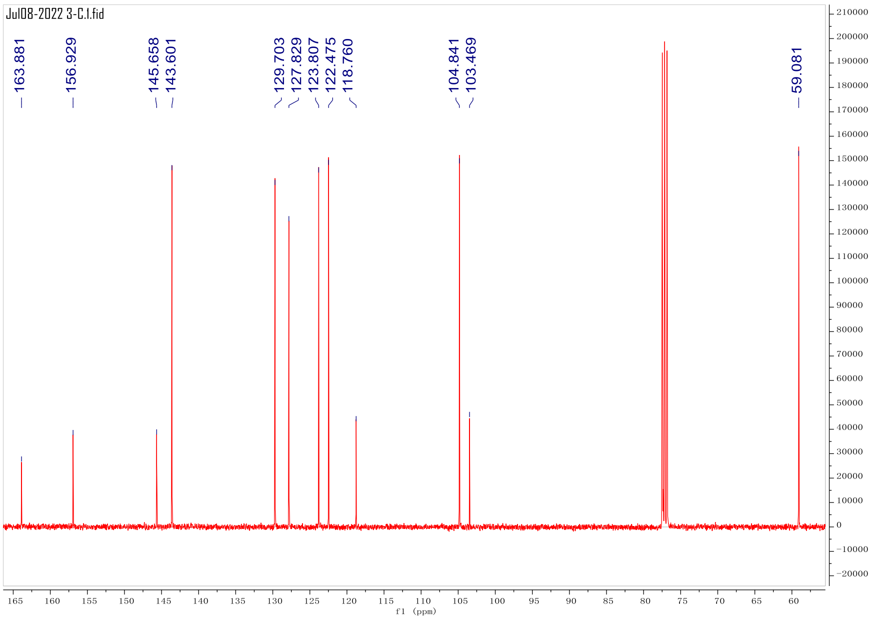

Figure S18. ^{13}C NMR spectrum of compound **6** in $CDCl_3$

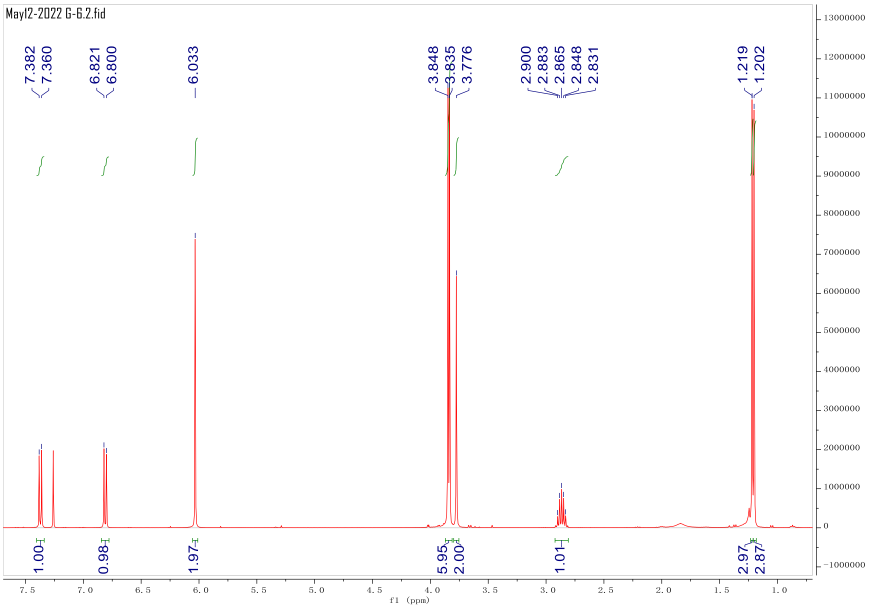

Figure S19. ^{1}H NMR spectrum of compound **7** in $CDCl_3$

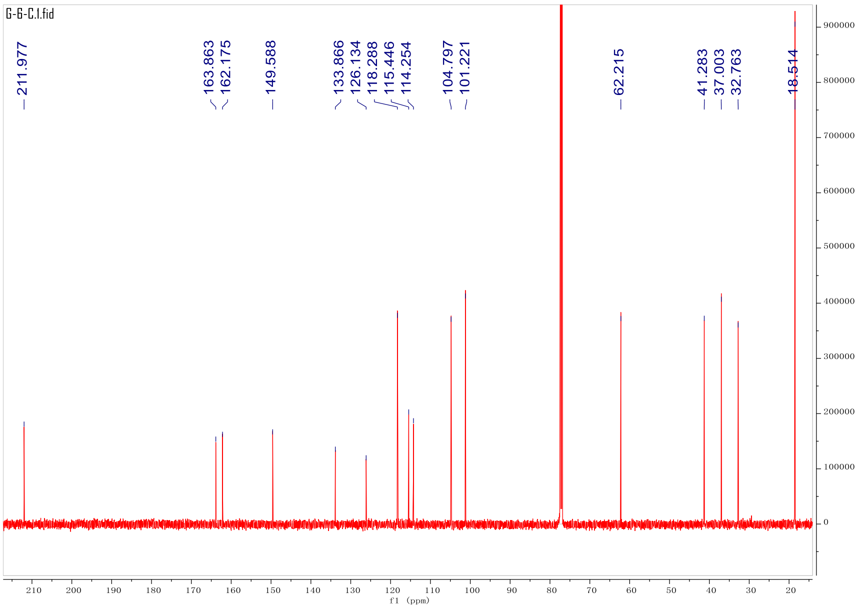

Figure S20. ^{13}C NMR spectrum of compound **7** in $CDCl_3$

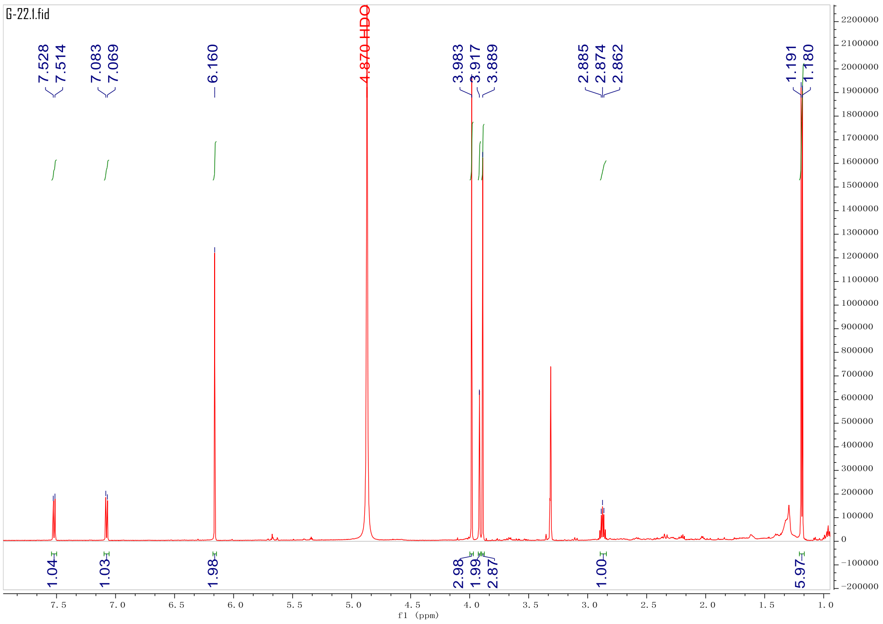

Figure S21. ^{1}H NMR spectrum of compound **8** in CD_3OD

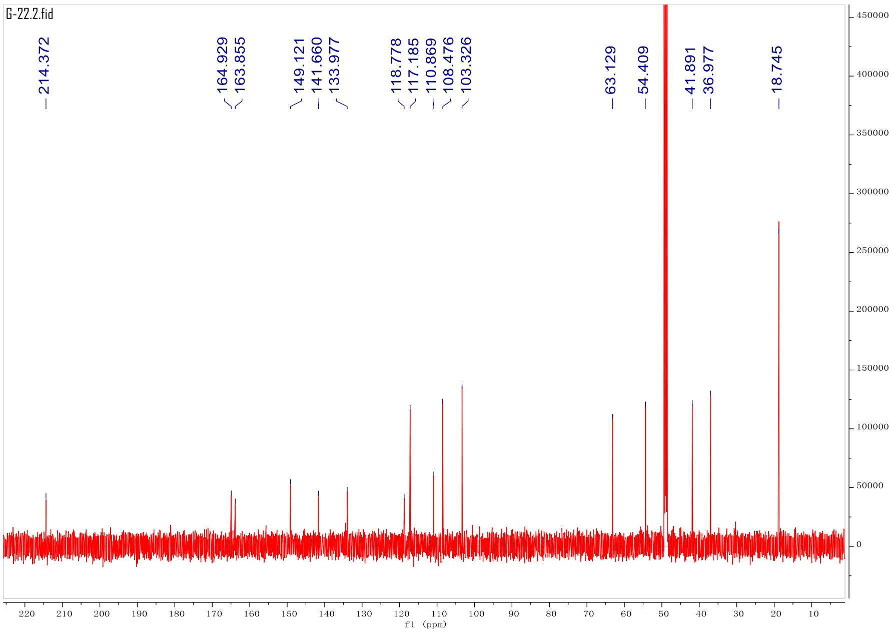

Figure S22. ^{13}C NMR spectrum of compound **8** in CD_3OD

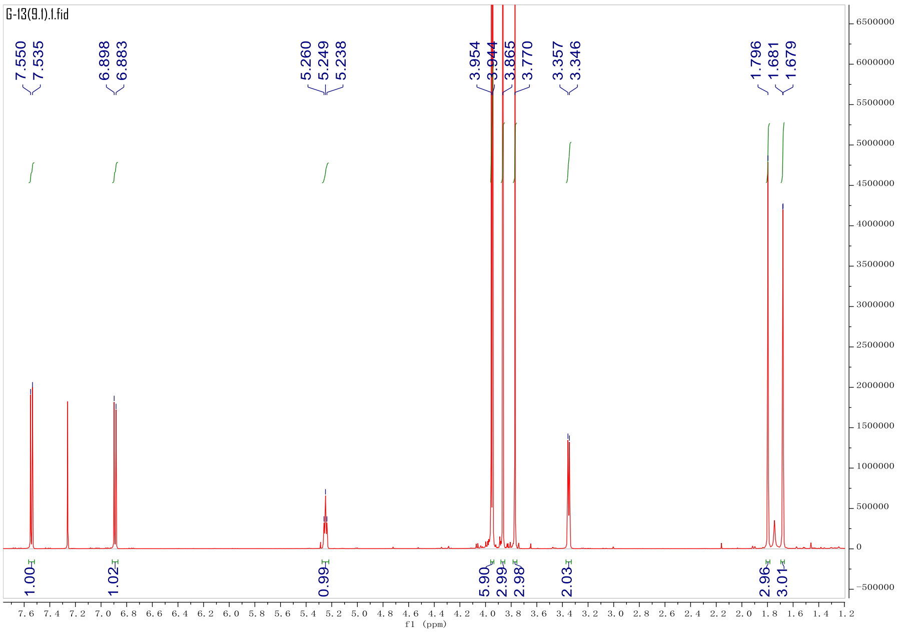

Figure S23. ^{1}H NMR spectrum of compound **9** in $CDCl_3$

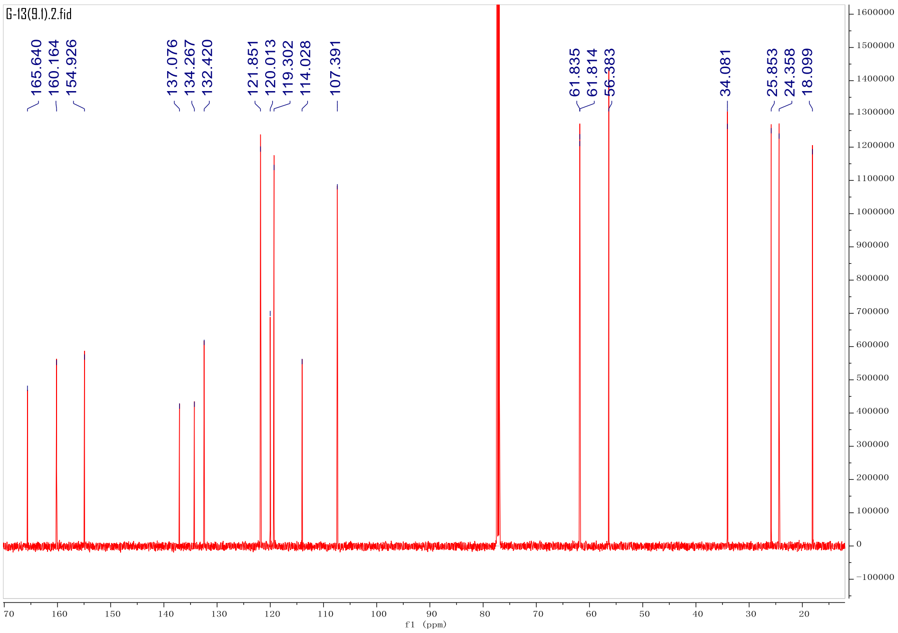

Figure S24. ^{13}C NMR spectrum of compound **9** in $CDCl_3$

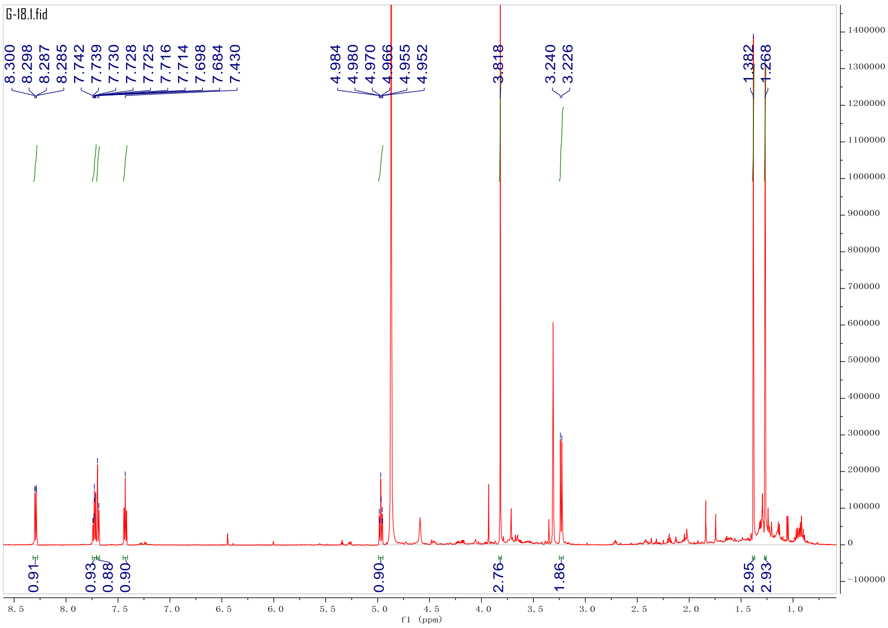

Figure S25. ^{1}H NMR spectrum of compound **10** in CD_3OD

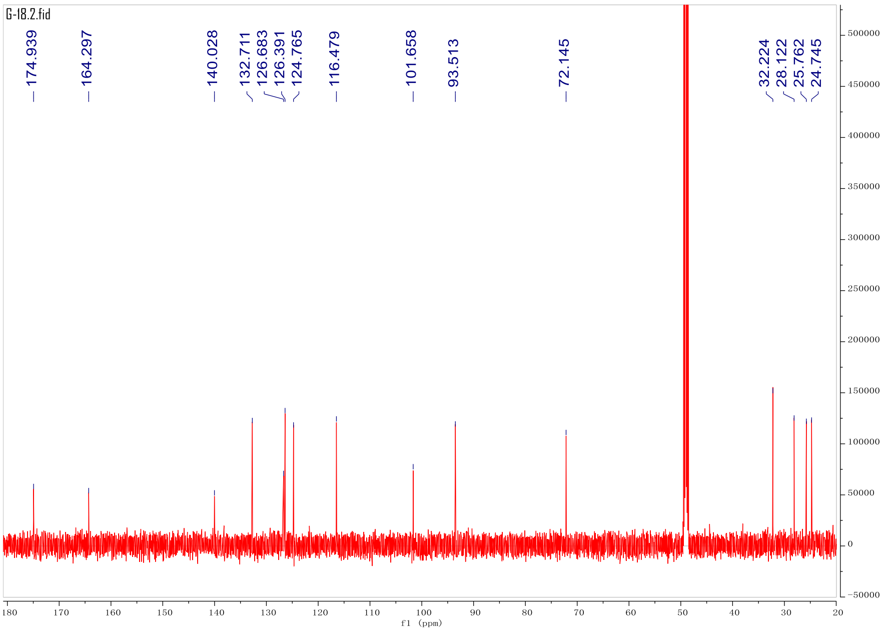

Figure S26. ^{13}C NMR spectrum of compound **10** in CD_3OD

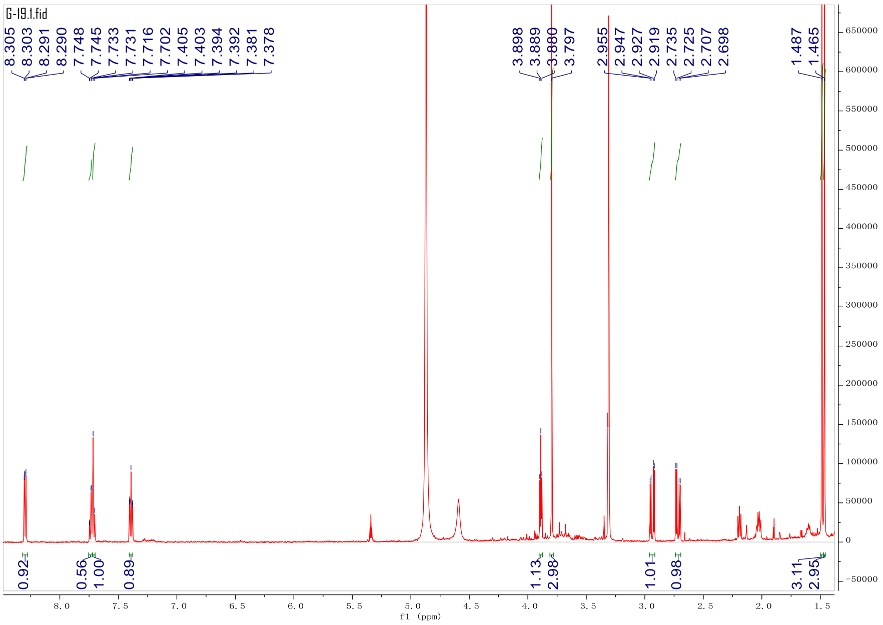

Figure S27. ^{1}H NMR spectrum of compound **11** in CD_3OD

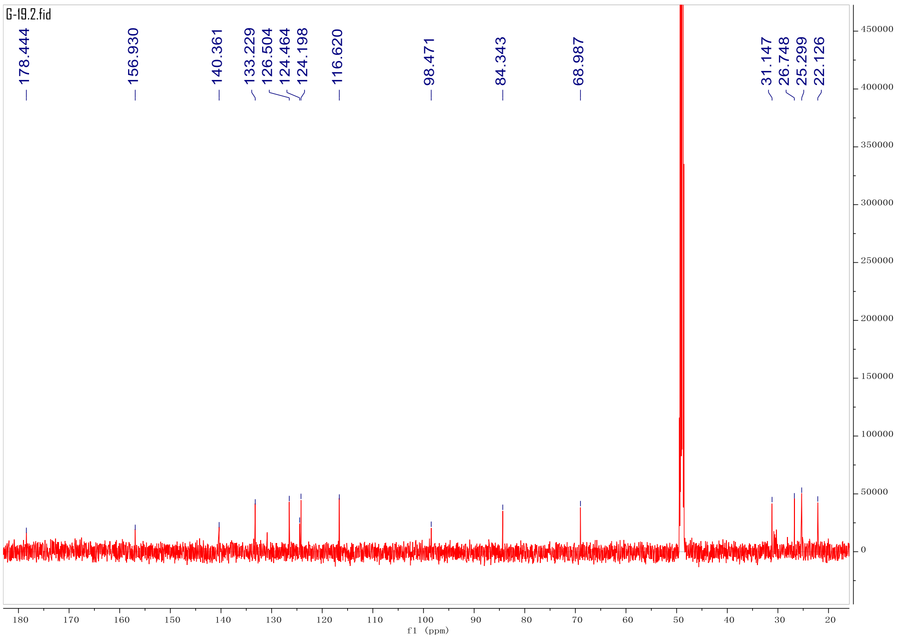

Figure S28. ^{13}C NMR spectrum of compound **11** in CD_3OD

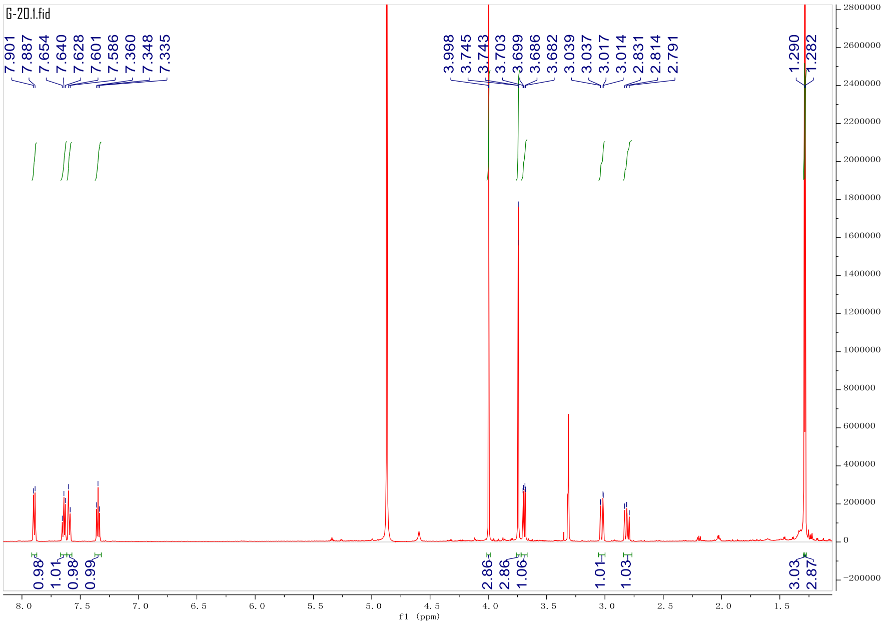

Figure S29. ^{1}H NMR spectrum of compound **12** in CD_3OD

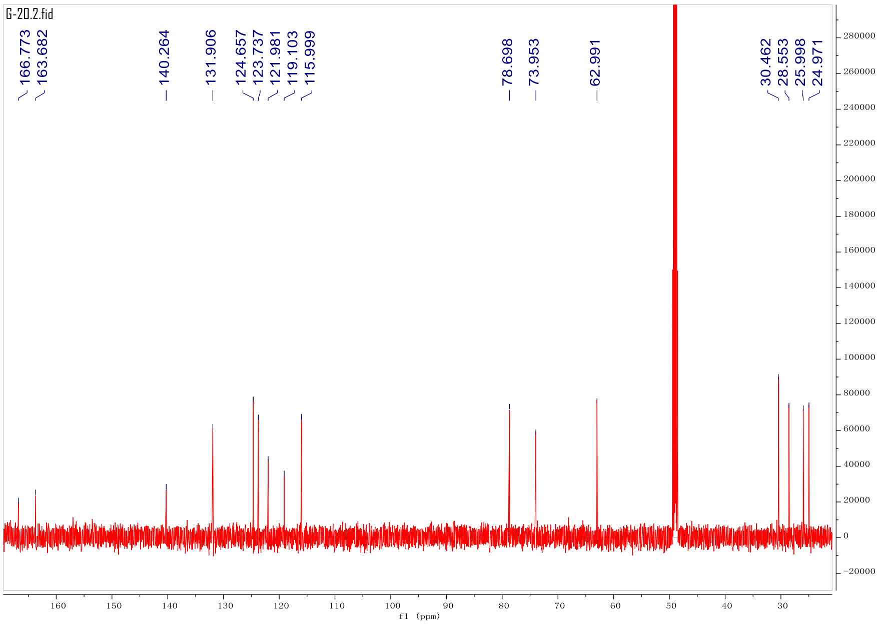

Figure S30. ^{13}C NMR spectrum of compound **12** in CD_3OD

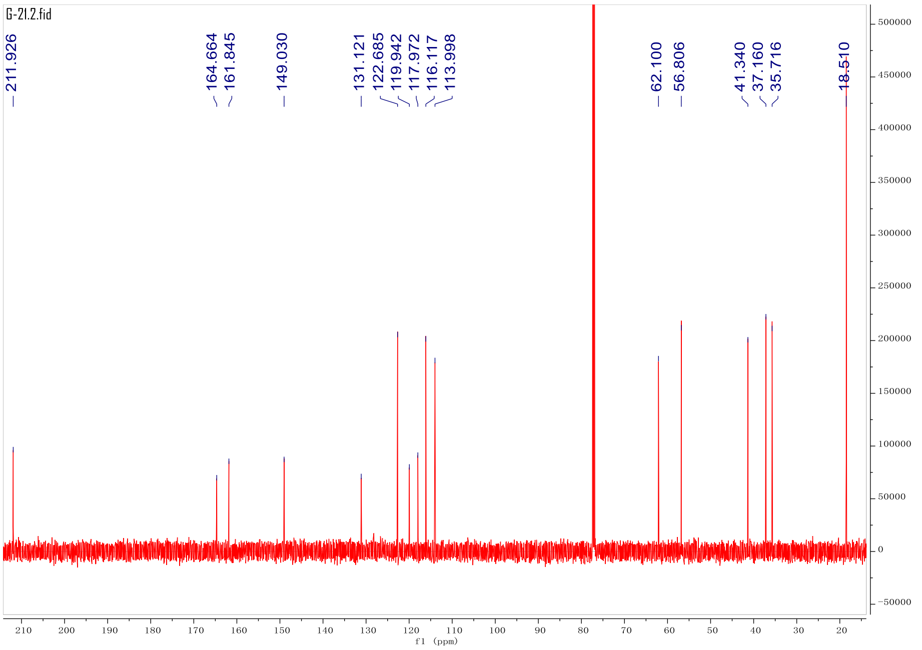

Figure S31. ^{1}H NMR spectrum of compound **13** in $CDCl_3$

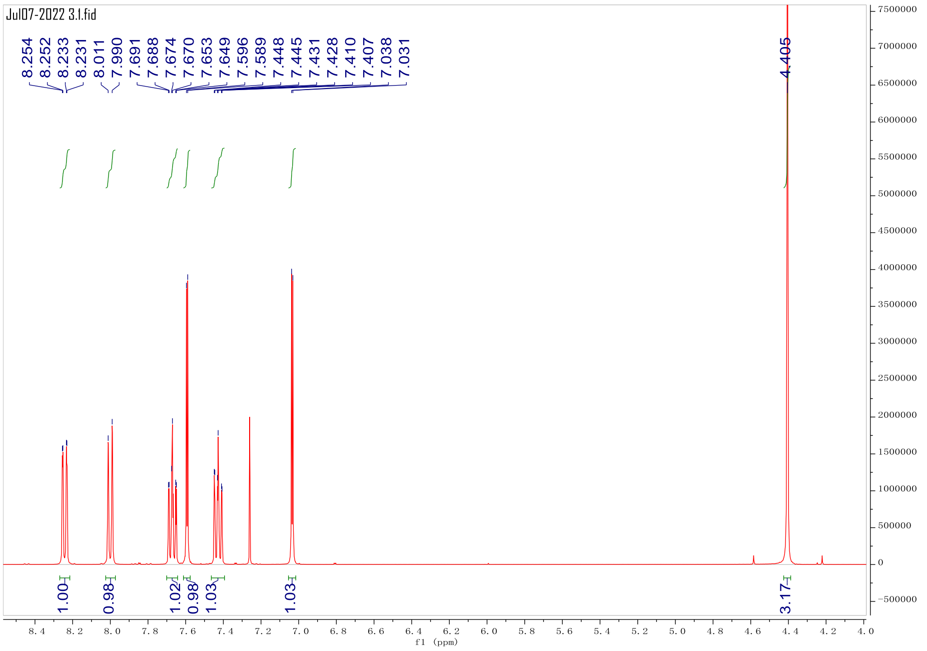

Figure S32. ^{13}C NMR spectrum of compound **13** in $CDCl_3$

ECD calculation details

The conformations of the isomers of compound **1** was generated by iMTD-GC method embedded in Crest program [1]. Two conformations with the root-mean-square (RMS) distance and energy deviation of 0.5 Å and 0.25 kcal/mol, respectively, were considered as duplicates and one of them was removed. Density functional theory calculations were performed with the Gaussian 09 package. ECD spectra were calculated by the TDDFT methodology at the B3LYP/def2TZVP utilizing IEFPCM in methanol. The final ECD spectra were simulated by averaging the spectra of lowest energy conformers according to the Boltzmann distribution theory and their relative Gibbs free energy (ΔG).

[1]. Pracht, P.; Bohle, F.; Grimme, S. Automated Exploration of the Low-Energy Chemical Space with Fast Quantum Chemical Methods. *Phys. Chem. Chem. Phys.* **2022**, *22*, 7169–7192.

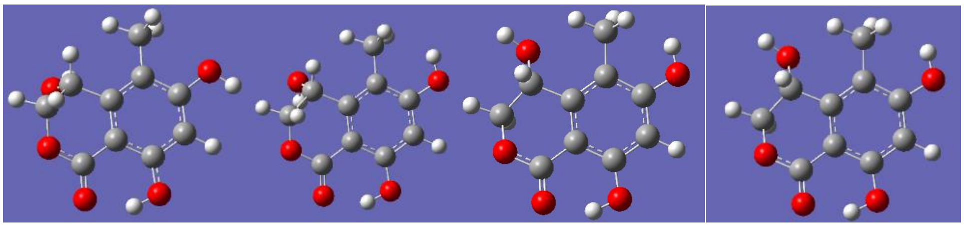

58.83% 38.94% 1.66% 0.57%

Optimized geometries of isomers of **1** at B3LYP/6-311G(d) level in methanol**.**

Figure S33. ECD calculation details of compound **1**
